# Epigenetics Identifier screens reveal regulators of chromatin acylation and limited specificity of acylation antibodies

**DOI:** 10.1038/s41598-021-91359-0

**Published:** 2021-06-17

**Authors:** Leonie Kollenstart, Sophie C. van der Horst, Kees Vreeken, George M. C. Janssen, Fabrizio Martino, Hanneke Vlaming, Peter A. van Veelen, Fred van Leeuwen, Haico van Attikum

**Affiliations:** 1 Department of Human Genetics, Leiden University Medical Center, Einthovenweg 20, 2333 ZC Leiden, The Netherlands; 2 Center for Proteomics and Metabolomics, Leiden University Medical Center, Albinusdreef 2, 2333 ZA Leiden, The Netherlands; 3 Centro de Investigaciones Biológicas (CIB), Consejo Superior de Investigaciones Científicas (Spanish National Research Council), (CSIC), Ramiro de Maeztu 9, 28040 Madrid, Spain; 4 Division of Gene Regulation, Netherlands Cancer Institute, Plesmanlaan 121, 1066 CX Amsterdam, The Netherlands; 5 Department of Medical Biology, Amsterdam UMC, University of Amsterdam, 1105 AZ Amsterdam, The Netherlands

**Keywords:** Epigenetics, Histone post-translational modifications, Acetyltransferases

## Abstract

The collection of known posttranslational modifications (PTMs) has expanded rapidly with the identification of various non-acetyl histone lysine acylations, such as crotonylation, succinylation and butyrylation, yet their regulation is still not fully understood. Through an unbiased chromatin immunoprecipitation (ChIP)-based approach called Epigenetics-IDentifier (Epi-ID), we aimed to identify regulators of crotonylation, succinylation and butyrylation in thousands of yeast mutants simultaneously. However, highly correlative results led us to further investigate the specificity of the pan-K-acyl antibodies used in our Epi-ID studies. This revealed cross-reactivity and lack of specificity of pan-K-acyl antibodies in various assays. Our findings suggest that the antibodies might recognize histone acetylation in vivo, in addition to histone acylation, due to the vast overabundance of acetylation compared to other acylation modifications in cells. Consequently, our Epi-ID screen mostly identified factors affecting histone acetylation, including known (e.g. *GCN5, HDA1*, and *HDA2*) and unanticipated (*MET7*, *MTF1, CLB3,* and *RAD26*) factors*,* expanding the repertoire of acetylation regulators. Antibody-independent follow-up experiments on the Gcn5-Ada2-Ada3 (ADA) complex revealed that, in addition to acetylation and crotonylation, ADA has the ability to butyrylate histones. Thus, our Epi-ID screens revealed limits of using pan-K-acyl antibodies in epigenetics research, expanded the repertoire of regulators of histone acetylation, and attributed butyrylation activity to the ADA complex.

## Introduction

In the eukaryotic nucleus, DNA is coiled around histone proteins to form nucleosomes, making up a higher-order structure called chromatin. Chromatin can be regulated by posttranslational modifications (PTMs), which are integral i﻿n processes such as gene transcription and DNA damage repair^1–3^. In recent years, the collection of known PTMs has substantially increased through the use of various mass spectrometry-based approaches. Among these PTMs are the non-acetyl histone lysine acylations such as crotonylation, succinylation and butyrylation^4–8^. Structurally, these modifications highly resemble acetylation. However, their hydrocarbon chains are longer, resulting in a bulkier structure, and some have a different charge. These non-acetylation PTMs, similar to acetylation, have been linked to transcriptional activity^9–13^. Particularly, the identification of writers and erasers of these PTMs has provided insight into their regulation. For example, histone acetyltransferases (HATs) like Gcn5, Esa1, MOF and p300 are all writers of crotonylation^12–14^, whereas class I histone deacetylases (HDACs) possess decrotonylation activity^13, 15–18^. While the regulation of non-acetyl acylations is still not fully understood, it has become apparent that their functionality is linked to metabolism^10, 18–22^. For example, crotonylation by Gcn5 and Esa1 is affected by the cellular uptake of external crotonate, leading to various transcriptional changes^13^. Moreover, during the yeast metabolic cycle (YMC), the increase in H3K9 crotonylation is linked to the repression of pro-growth genes^10^.

To further improve our understanding of various histone acylations, we performed an unbiased genome-wide screen, named Epigenetics-IDentifier (Epi-ID), to identify regulators of crotonylation, succinylation and butyrylation. Epi-ID has been successfully applied to identify regulators of histone H3K79 methylation and histone turnover^23, 24^. It employs chromatin immunoprecipitation (ChIP) to monitor the chromatin status of a barcoded locus in yeast mutants in a high-throughput manner^25^. Here, we applied Epi-ID to identify regulators of histone crotonylation, succinylation and butyrylation by screening thousands of mutant yeast strains using pan-K-acyl-recognizing antibodies^5, 6, 26^. The screens identified a variety of potential regulators, which were highly similar between all acylations. This prompted us to further investigate the specificity of the antibodies used in these screens. Remarkably, cross-recognition of epitopes by the pan-K-acyl-recognizing antibodies was observed in various assays in yeast and human cells. Given these findings, we hypothesized that the regulators identified in our Epi-ID screens are either regulators of histone acetylation or histone acylation. To discriminate between these possibilities, we further investigated the Gcn5-Ada2-Ada3 (ADA) complex, a writer for acetylation and crotonylation and one of the strongest common hits in the Epi-ID screens^13^. Follow-up experiments, independent of antibody cross-reactivity, revealed a novel butyrylation activity of the ADA complex. In addition, we also validated the effects of various potential acetylation regulators. Thus, the Epi-ID screens revealed pan-K-acyl antibody aspecificity, regulators of histone acetylation, and a new role for the ADA complex in histone butyrylation.

## Results

### Epi-ID screens for regulators of histone acylation in yeast

**Figure 1:**
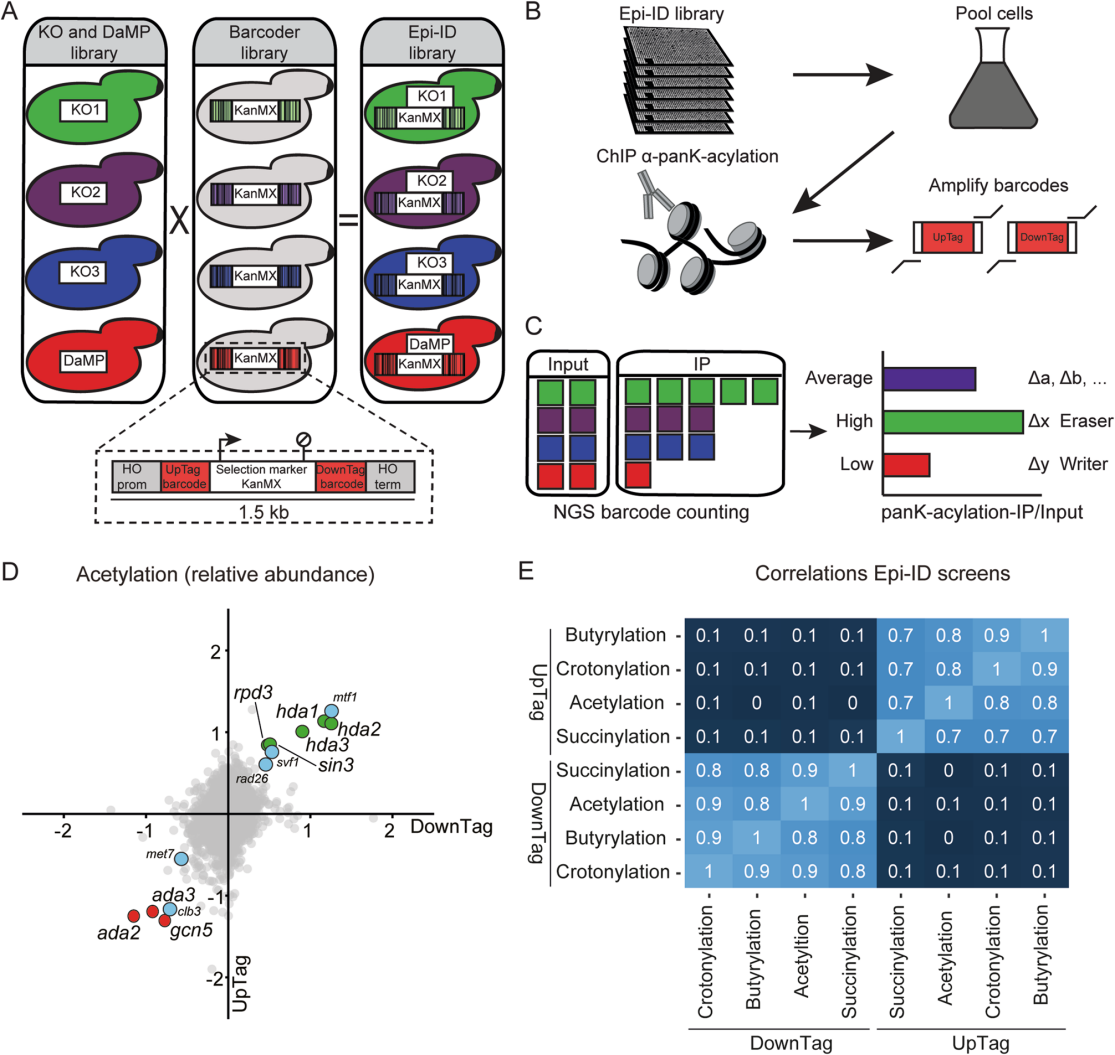
Epi-ID identifies regulators of chromatin. (**A**) Construction of the Epi-ID library. Knock-out and DAmP libraries of yeast mutants were crossed to a Barcoder library of yeast strains using SGA technology. Each strain in the Barcoder library contains a KanMX selection gene flanked by unique 20 bp UpTag and 20 bp DownTag barcodes integrated at the *HO* locus. (**B**) Outline of Epi-ID. Strains from the Epi-ID library were pooled, grown in liquid medium and subjected to ChIP using pan-K-acyl antibodies. The barcodes were amplified from ChIP and input DNA. (**C**) Next-generation sequencing of barcodes. Barcodes counted in immpunoprecipitates were normalized to counts in input. (**D**) Outcome of Epi-ID for acetylation on approximately 6500 mutant strains. Scatter plots present log2-transformed acetyl ChIP/input ratios normalized to H3 ChIP/input ratios for UpTag versus DownTag. Averages of two independent Epi-ID screens are shown. Each dot represents a single mutant strain. Mutants of the ADA (red), RPD3 (green), HDA1 (green) complexes and newly identified mutants selected for follow-up (blue) are highlighted. (**E**) Spearman correlations plotted for all Epi-ID screens, unnormalized for H3.

To find regulators of crotonylation, succinylation and butyrylation, we used an unbiased approach in yeast, called Epi-ID^23^, to identify mutants that affect the acylation status of chromatin. Epi-ID interrogates the chromatin status on DNA barcodes in yeast mutants through ChIP on pooled cells. In addition to a collection of ~ 4700 yeast deletion mutants^27^ that we employed previously^23^, we used a collection of ~ 2000 Decreased Abundance by mRNA Perturbation (DAmP) alleles for essential genes^28^. These yeast mutants were crossed with the Barcoder yeast library using Synthetic Genetic Array (SGA) technologies to create mutants containing a pair of unique barcodes, the UpTag and DownTag barcodes, surrounding a KanMX marker gene at the *HO* locus (Fig. 1A)^29^. This system enabled us to introduce in each yeast mutant a barcode pair at a common chromatin locus.

We confirmed pan-K-acyl antibody recognition at the barcodes by performing ChIP coupled to quantitative (q)PCR on wild-type yeast cells with pan-K-recognizing antibodies for crotonylation, succinylation and butyrylation. ChIP signals were enriched at both barcodes for all three antibodies (Supplementary Fig. 1A–C). The signals were higher in the promoter-proximal UpTag compared to the DownTag located at the terminator, suggesting higher crotonylation, succinylation and butyrylation levels in promoter regions as compared to terminator regions. This relative enrichment at the promoter is reminiscent of acetylation and other acylation marks that are linked to transcription activity^9, 10, 30^.

Next, the collection of barcoded yeast mutants was pooled and subjected to ChIP using pan-recognizing acyl-K-antibodies, including antibodies recognizing pan-K-acetylation and histone H3 as controls. Barcodes from input and immunoprecipitated DNA samples were amplified by PCR and subsequently identified and counted using Next-Generation Sequencing (Fig. 1B,C). The amount of histone modification present in a single yeast mutant is reflected by the abundance of a barcode in the immunoprecipitate compared to that in the input (ChIP/input) (Fig. 1D). For all acyl modifications, the mean abundance in two independent Epi-ID screens was plotted for UpTag and DownTag, normalized to H3 occupancy (Fig. 1D and Supplementary Fig. 1D–F). All barcode abundance data can be found in Supplementary Information 2. Remarkably, the screens for the different acylation marks showed very high levels of correlation (Fig. 1E), suggesting that the various mutants affect these acylation modifications similarly. Indeed, several common outliers (> 1.5-fold change) could be detected over the background. Outliers included three members of the ADA complex, namely the histone acetyltransferase Gcn5 and the Ada2 and Ada3 subunits, as potential positive regulators, and core components of the Rpd3/Sin3 and Hda1/2/3 deacetylase complexes as potential negative regulators of crotonylation, succinylation and butyrylation (Fig. 1D and Supplementary Fig. 1D–F). For ADA and Rpd3 complexes this could be partially explained since both contain catalytic activities involved in (de)acetylation and (de)crotonylation, while they are not known to (de)butyrylate or (de)succinylate^13, 15, 17^. In addition, the genome-wide localization of crotonylation and butyrylation marks strongly correlates with that of several histone acetylation marks, suggesting they may perform biologically similar functions^14, 18, 31^. This suggests that our Epi-ID screens may serve as a resource for the identification and further characterization of regulators of histone acylation.

### Pan-K-acyl antibody validation using dot-blot assays

**Figure 2:**
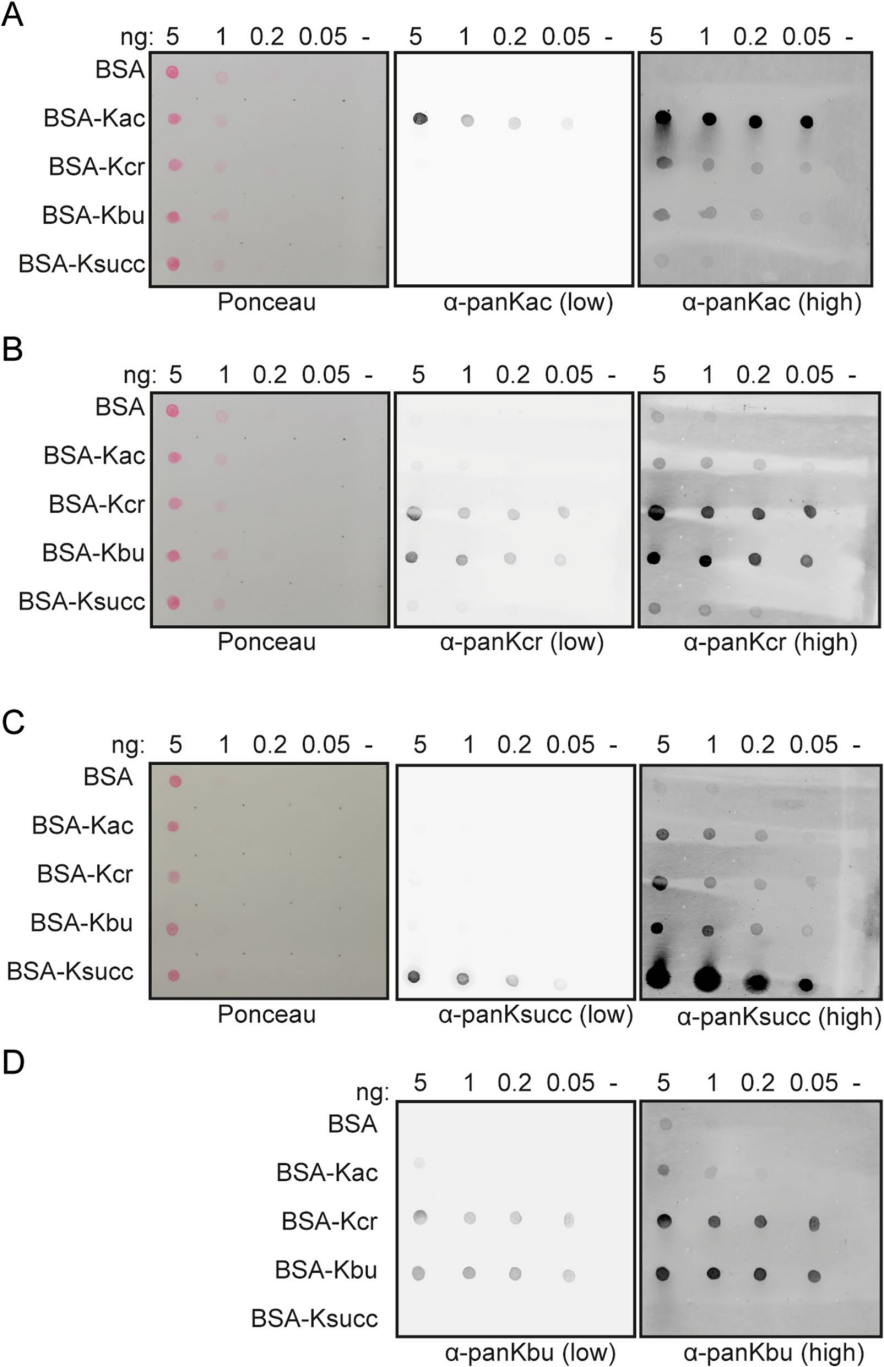
Dot-blots of modified BSA to test pan-K-acyl antibody specificity. (**A**–**D**) Immunoblot analysis of serially diluted and differently modified BSA with the indicated antibodies. Ponceau staining in (**C**) is for both succinylation and butyrylation blots.

The fact that our Epi-ID screen results using different pan-K-acyl-recognizing antibodies were highly correlated (Fig. 1E), prompted us to examine the validity of these antibodies in recognizing different acyl modifications in various assays. First, we tested the specificity of the pan-K-acyl-recognizing antibodies in dot-blot assays using in vitro acylated BSA. Mass spectrometry analysis confirmed efficient acylation of BSA, albeit that the degree of modification was somewhat lower for succinyl-BSA (Table 1). Low exposure images of the dot blots suggested that the pan-K-acetyl and pan-K-succinyl antibodies robustly recognize acetyl-BSA and succinyl-BSA, respectively (Fig. 2A,C), whereas pan-K-crotonyl and pan-K-butyryl antibodies were unable to differentiate between crotonyl-BSA and butyryl-BSA (Fig. 2B,D). Moreover, high exposure images of the blots revealed that all antibodies recognize the different versions of modified BSA to a certain extent over non-modified BSA (Fig. 2A–D). Similar results were obtained with antibodies from older lot numbers (Supplementary Fig. 2A–D). Therefore, while pan-K-acetyl and pan-K-succinyl are mostly specific, the pan-K-crotonyl and pan-K-butyryl antibodies show cross-reactivity to their respective targets likely due to their highly similar structures with identical carbon-chain lengths.

**Table 1:** Spectral counts of acyl-modifications in BSA.

Sample	K-acetyl	K-crotonyl	K-butyryl	K-succinyl	Total
Unmodified BSA	1	0	0	0	679
Acetyl-BSA	316	0	0	0	436
Crotonyl-BSA	4	97	0	1	109
Succinyl-BSA	0	0	1	79	244
Butyryl-BSA	1	1	270	0	420

### Pan-K-acyl antibody validation using western blot assays

**Figure 3:**
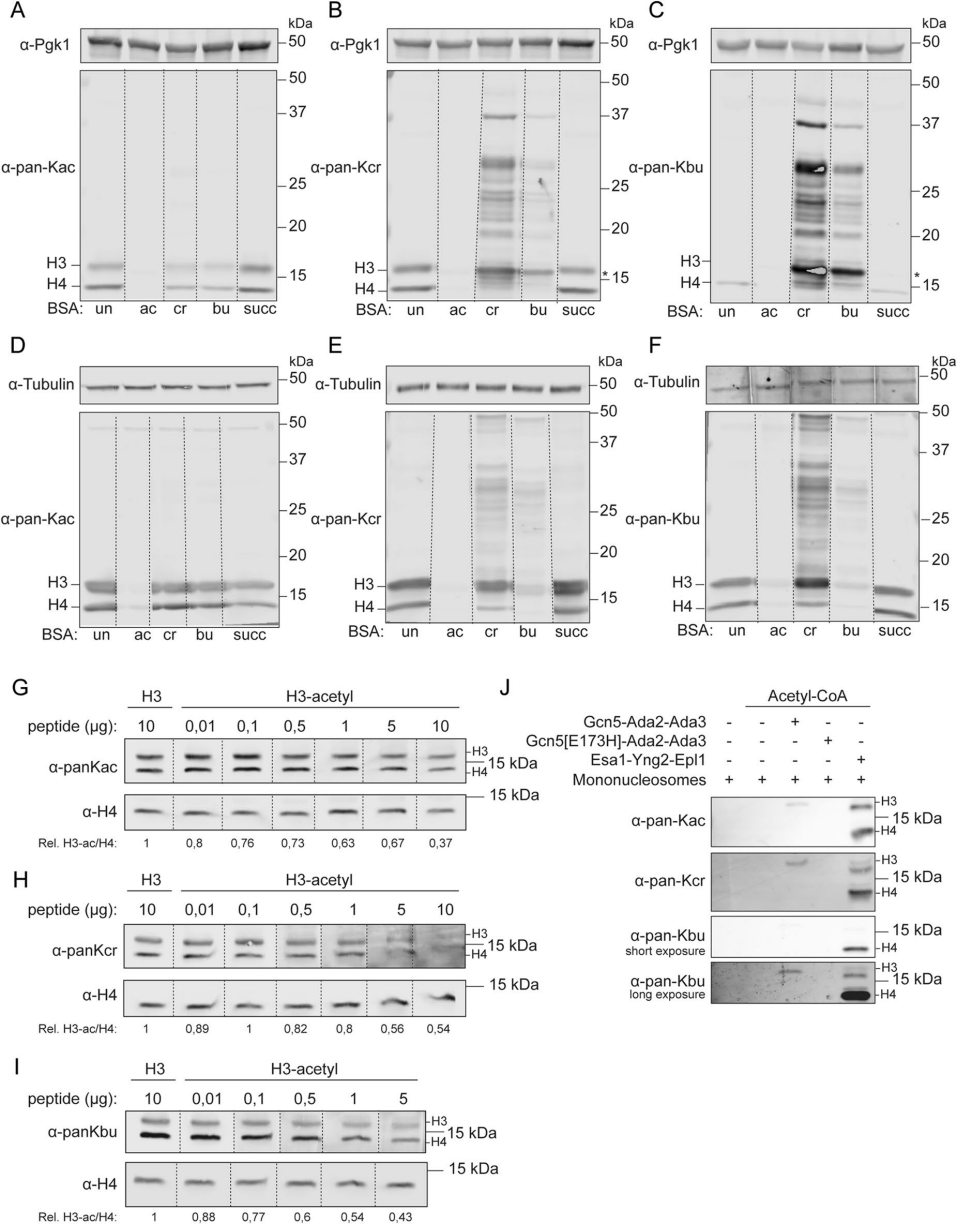
Western blot analysis of pan-K-acyl antibodies with BSA competitors on yeast and HeLa cell extracts. Whole-cell extracts of wild-type yeast cells (**A**–**C**) and HeLa cells (**D**–**F**) were immunoblotted with the indicated antibodies in the presence of (modified) BSA. All lanes contain the same yeast or HeLa whole-cell extract. 0.5 µg BSA per microliter of antibody was added. Asterisk highlights background band. Whole-cell extracts of wild-type yeast cells (**G**–**I**) were immunoblotted with the indicated antibodies in the presence of (acetylated) H3 peptide. All lanes contain the same whole-cell extract. Below the blots is a quantification of the acylation signal over H4 of at least two independent experiments. (**J**) Western blot analysis of in vitro histone acetyltransferase assay with recombinant wild-type and catalytic-dead (Gcn5-E173H) ADA complex and wild-type Piccolo NuA4 complex immunoblotted with the indicated antibodies.

Since all antibodies showed a modest recognition of all forms of acylated BSA, we continued testing their specificity in whole-cell extracts, in which each acylation modification occurs at a different abundance. Particularly, the modest recognition of acetylation by the non-acetyl antibodies may affect their specificity in whole-cell extracts as acetyl marks are generally highly abundant compared to the non-acetylation marks. Since the pan-K-acyl antibodies are often used in western blot experiments^9, 12, 15, 32–35^, we determined the specificity of these antibodies in western blot competition assays using whole-cell extracts from wild-type yeast cells and human (HeLa) cells. Individual lanes of the blots were incubated with either of the pan-K-acyl antibodies in combination with the different modified versions of BSA. For both the yeast and human cell extracts, the pan-K-acetylation signal on histones was completely outcompeted by acetyl-BSA, but also to a lesser extent by crotonyl-BSA and butyryl-BSA (Fig. 3A,D). Strikingly, when using pan-K-crotonyl and pan-K-butyryl antibodies, crotonylation and butyrylation signals on histones were fully outcompeted by acetyl-BSA, again showing that these two antibodies cross-react with acetylation. Crotonyl-BSA and butyryl-BSA partially removed the histone signals but with the concomitant appearance of background bands (Fig. 3B,C,E,F). Apparently, the minor recognition of acetyl-BSA by the crotonyl and butyryl antibodies, as seen on the dot-blot (Figs. 2 and S2), is sufficient to outcompete all signals produced by these antibodies in western blot assays. The pan-K-crotonyl and pan-K-butyryl antibodies are purified polyclonal antibodies and their signals should, therefore, be fully depleted in the presence of its epitope. Currently, we cannot fully explain the appearance of the higher molecular weight bands and speculate that they might be due to acyl moieties from the BSA preparations reacting with proteins on the blot. The pan-K-succinyl antibody could not detect succinylation in western blot analysis.

Next, we tested whether K9- and K14-acetylated H3 peptides (residues 1–21) could also compete with the pan-K-acyl antibodies. We found that upon incubation with acetyl-H3, up to 60% of the acetylation signal on H3 decreased following western blot analysis of whole-cell extracts from wild-type yeast cells, suggesting acetyl-H3 is a less competitive epitope than acetyl-BSA (Fig. 3G). Similarly, signals for histone crotonylation and butyrylation were also decreased after incubation with the acetyl-H3 peptide, indicating cross-reactivity between histone acetylation and these non-acetyl antibodies (Fig. 3H,I).

Finally, we tested whether the non-acetyl antibodies recognize acetylation marks generated by Gcn5 and Esa1 on mononucleosomes in vitro. To this end, we employed previously purified core ADA complex (wild-type Gcn5, Ada2, Ada3; catalytic-dead Gcn5 (E173H), Ada2, Ada3), and Piccolo NuA4 complex (Esa1, Yng2, Epl1)^13^. These complexes, except the catalytically-dead ADA complex, were proficient in acetylating nucleosomes (Fig. 3J). Acetylation by both ADA and Piccolo NuA4 complexes, by solely providing acetyl-CoA as co-factor, was recognized by pan-K-crotonyl and pan-K-butyryl antibodies (Fig. 3J). Thus, the pan-K-crotonyl and pan-K-butyryl antibodies do not specifically recognize their targets in western blot analysis and are capable of recognizing histone acetylation.

### Pan-K-acyl antibody validation using immunofluorescence assays

**Figure 4:**
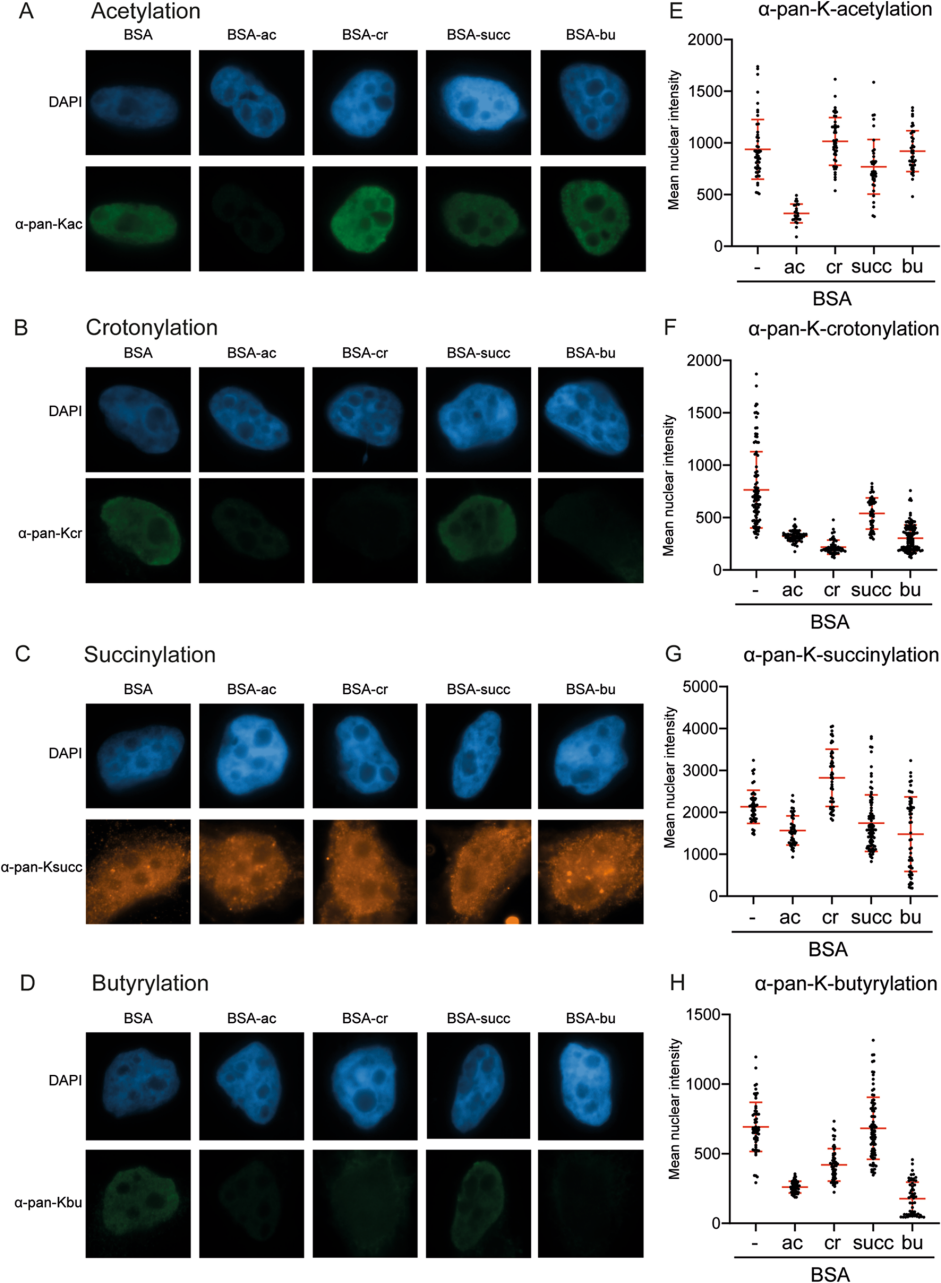
Immunofluorescence assays using pan-K-acyl antibodies with BSA competitors on HeLa cells. (**A**) Representative immunofluorescence images of HeLa cells showing nuclei stained with DAPI and pan-K-acetyl-recognizing antibodies against (**A**) acetylation, (**B**) crotonylation, (**C**) succinylation or (**D**) butyrylation in the presence of the indicated BSA competitors. (**E**–**H**) Quantification of data, representing the mean intensity of individual nuclei from (**A**–**D**), respectively. For each microliter of antibody, one microgram of BSA was added as competitor.

In addition to their application in western blot analysis, the pan-K-acyl antibodies have been previously used in immunofluorescence studies^12, 15, 36^. Therefore, we performed immunofluorescence on HeLa cells using the modified versions of BSA as competitors during the incubation with primary antibodies and quantified the nuclear intensity of individual cells. HeLa cells stained with the pan-K-acyl antibodies showed pan-nuclear staining (Fig. 4A–D), as reported previously^12, 15^. The signal generated with the pan-K-acetyl antibody signal was reduced in the presence of the acetyl-BSA competitor, but not with the other modified forms of BSA, in agreement with the western blot analysis (Fig. 4A,E). However, for both crotonylation and butyrylation, pan-nuclear staining was reduced by acetyl-BSA, crotonyl-BSA and butyryl-BSA (Fig. 4B,D,F,H). On the contrary, the pan-K-succinyl antibody generated a signal, but this could not be outcompeted by any of the acyl-BSA competitors (Fig. 4C,G). Therefore, we conclude that the pan-K-crotonyl, pan-K-succinyl and pan-K-butyryl antibodies tested here do not function as a specific agent in immunofluorescence assays.

### Pan-K-acyl antibody validation using ChIP-qPCR assays

**Figure 5:**
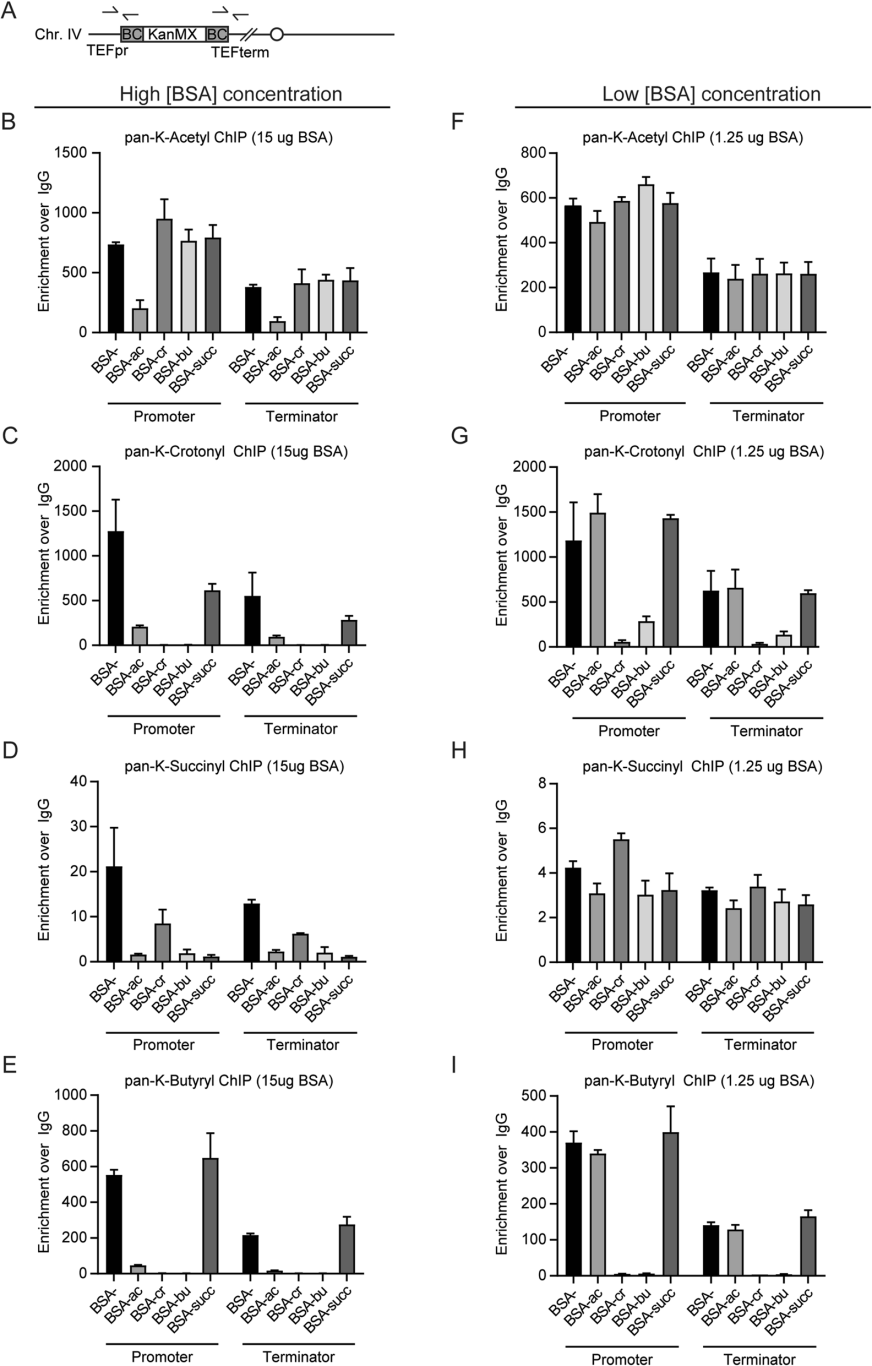
ChIP-qPCR analysis of pan-K-acyl antibodies with BSA competitors in yeast. (**A**) Overview of the KanMX cassette integrated at the *HO* locus on chromosome IV. Binding sites of primers at the TEF promoter and TEF terminator used for ChIP-qPCR are indicated. These sites are located 43 and 119 bases away from the barcoded region, respectively. (**B**–**I**) ChIP-qPCR analysis of TEF promoter and TEF terminator regions of the KANMX selection gene at the *HO* locus in wild-type yeast using the indicated pan-K-acyl antibodies in combination with high and low amounts of the indicated BSA competitors. In high BSA conditions, 6 µg of BSA was added per microliter of antibody. In low BSA conditions, 0.5 µg of BSA was added per microliter of antibody. Data represent the mean fold enrichment over background (IgG) from two independent experiments + s.e.m.

Since we used the pan-K-acylation antibodies during Epi-ID screening in a ChIP-based method, we determined by ChIP-qPCR the extent to which the pan-K-acyl antibodies recognize the different acylation marks at the barcodes present at the *HO* locus. Since no yeast mutant is known wherein a specific acylation modification is completely “erased”, we again used the differently modified versions of BSA as competitors. To this end, various forms of BSA were added to the chromatin extracts prior to the immunoprecipitations. ChIP signals generated at the UpTag and DownTag barcodes by the pan-K-acetyl antibody (Fig. 5A) were only outcompeted by acetyl-BSA (Fig. 5B), indicating specificity of the acetylation antibody in ChIP-qPCR assays (Fig. 5B). In contrast, both crotonyl-BSA and butyryl-BSA severely reduced the signals generated by the pan-K-crotonyl and pan-K-butyryl antibodies, confirming that these antibodies recognize both crotonylation and butyrylation (Figs. 2D, 5C,E). Moreover, the addition of acetyl-BSA dramatically reduced the signals generated by the pan-K-crotonyl, pan-K-succinyl and pan-K-butyryl antibodies (Fig. 5C–E). Thus, the minor recognition of acetyl-BSA by the crotonyl, succinyl and butyryl antibodies, as seen on the dot-blots (Figs. 2 and S2), is sufficient to outcompete all signals produced by these antibodies in ChIP-qPCR assays (Fig. 5). This finding is problematic since the amount of histone acetylation exceeds the amount of the other non-acetyl acylations at least 200 times in eukaryotic cells^21^. We infer that pan-K-crotonyl, pan-K-succinyl and pan-K-butyryl antibody signals could arise from acetylation recognition.

To investigate this further, we repeated the ChIP assays with a minimal amount of BSA competitor. For antibodies that bind acetylation with low affinity, even low amounts of the true epitope will compete the antibody off the chromatin if most of the ChIP signal is due to cross-reactivity with acetylation. Indeed, adding sparse amounts of acetyl-BSA was not sufficient to compete with pan-K-acetylation antibody binding (Fig. 5F), suggesting this antibody binds acetylation in vivo. However, introducing a minimum amount of crotonyl-BSA or butyryl-BSA was sufficient to effectively reduce all signals generated by the pan-K-crotonyl and pan-K-butyryl antibodies (Fig. 5G,I), while no effect was observed when using the succinyl-BSA (Fig. 5G,I). This full depletion with sparse amounts of BSA suggests that the signals generated with pan-K-crotonyl and pan-K-butyryl antibodies originateding from acetylation rather than from other acylation modifications. No competition was seen with the pan-K-succinylation antibody, but considering all modified forms of BSA can compete with binding in higher concentrations, it suggests this antibody is binding acylation aspecifically (Fig. 5H)*.* Therefore, we conclude that the results of our Epi-ID screens do not accurately represent the effect of yeast mutants on the various acylation modifications, but most likely reflect their effect on acetylation.

### Epi-ID identifies potential new regulators of histone acetylation

**Figure 6:**
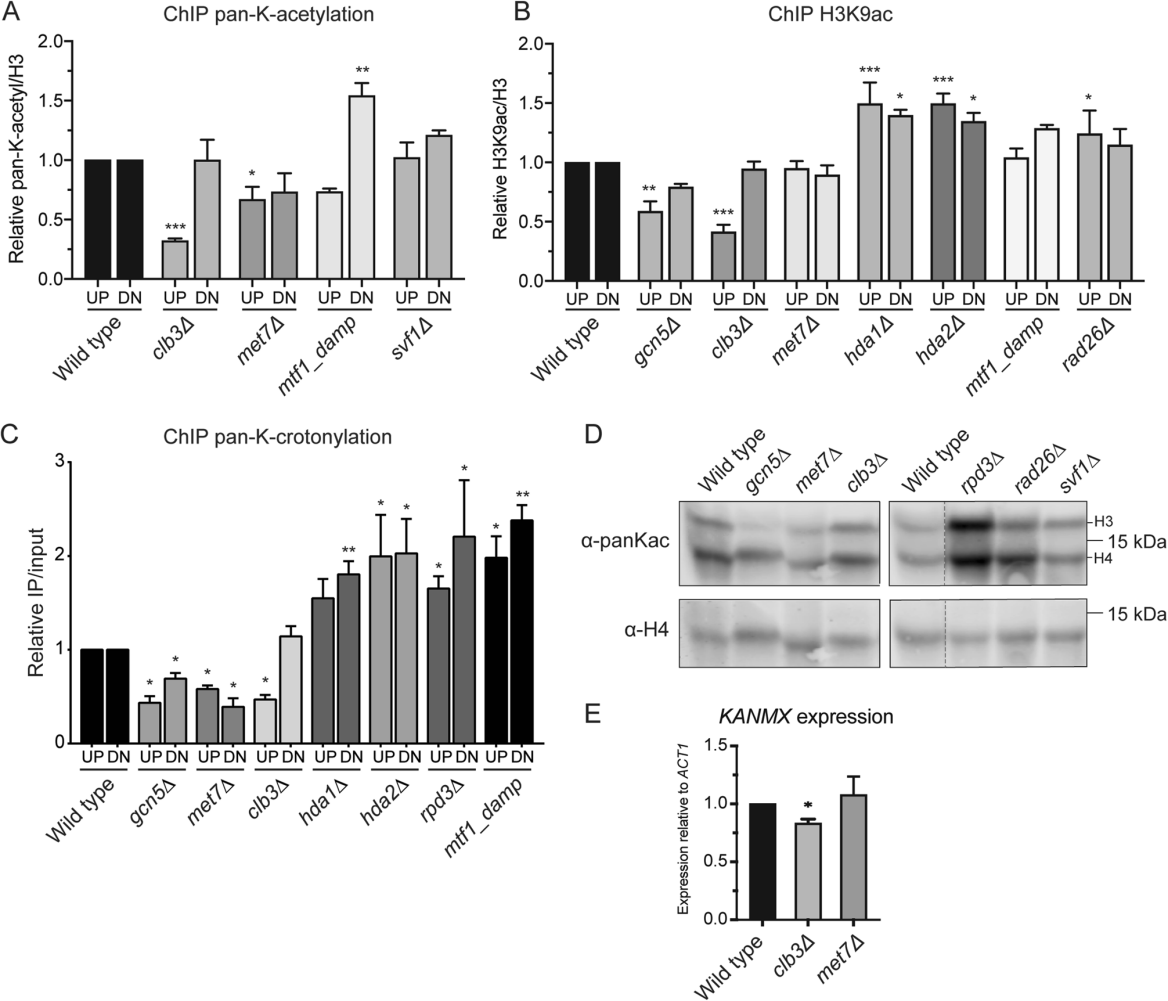
Epi-ID reveals potential regulators of acetylation and ADA complex displays histone butyryltransferase activity. (**A**–**C**) ChIP-qPCR analysis of pan-K-acetylation (**A**), H3K9ac (**B**) and pan-K-crotonylation (**C**) at UpTag and DownTag in a selection of hits from the Epi-ID screens. Averages of at least three independent experiments + s.e.m. are presented. Pan-K-acetylation and H3K9ac levels were normalized to H3 occupancy and signals at a telomeric region, and compared to wild-type (set to 1) using a one-sample T test for UpTag or DownTag. (**D**) Western blot analysis of global pan-K-acetylation in whole-cell extracts from the indicated yeast strains, dotted line indicates a cropped blot. All uncropped blots are shown in Supplementary Figure 5. (**E**) RT-qPCR analysis of *KANMX* expression in wild-type, *clb3Δ* and *met7Δ* strains. Expression is normalized to *ACT1*.

The Epi-ID screens identified various known and unanticipated factors that potentially affect histone acylation, particularly histone acetylation (Fig. 1D). To validate the Epi-ID screens, we examined the acetylation status of the barcodes in a selected panel of mutants using ChIP-qPCR. We selected candidates based on their common effect on both barcodes in all screens, showing at least a 1.5-fold change on average. Using this approach, we found that loss of *GCN5, MET7* and *CLB3,* but not of *SVF1,* resulted in a decrease in the pan-K-acetylation and/or H3K9 acetylation levels at the UpTag barcode, whereas loss of *HDA1, HDA2, MTF1* and *RAD26* led to an increase in the acetylation levels at both barcodes, confirming findings from the Epi-ID screen and showing the validity of the Epi-ID approach (Fig. 6A,B). For *MET7*, *CLB3* and *MTF1* these results were also confirmed on both barcodes using the pan-K-crotonylation antibody (Fig. 6C). To determine whether these genes and other top hits from the Epi-ID screens regulate histone acetylation levels throughout chromatin, we performed western blot analysis using the pan-K-acetyl antibody. This revealed that loss of *GCN5* strongly reduces histone acetylation, most prominently on histone H3, which is in line with its known role as an H3-specific HAT (Fig. 6D)^37, 38^. Interestingly, loss of *MET7,* but not that of *CLB3*, reduced histone acetylation, albeit that the effect was moderate when compared to that after *GCN5* loss (Fig. 6D). On the other hand, loss of *RPD3* ﻿lead to a strong increase in histone acetylation, which is in line with its known role as an HDAC^39^, whereas *RAD26* and *SVF1* moderately increased histone acetylation, indicating we can validate a global, but not a local effect of *SVF1*. Since gene expression correlates with histone acetylation^40, 41^, we investigated if changes in expression of the KanMX marker accompanied loss of acetylation on the barcodes in *CLB3* and *MET7* mutants. Indeed, loss of acetylation on the promoter region in *CLB3* mutants coincided with small but significant decrease of KanMX expression, while the expression was unaffected in *MET7* mutants (Fig. 6E). These results demonstrate that our Epi-ID screens provide a resource for the identification of known (*GCN5, HDA1, HDA2, RPD3*) and unanticipated *(MTF1, MET7*, *CLB3,* and *RAD26*) modulators of histone acetylation.

### Gcn5 is a writer for histone butyrylation in vitro

**Figure 7:**
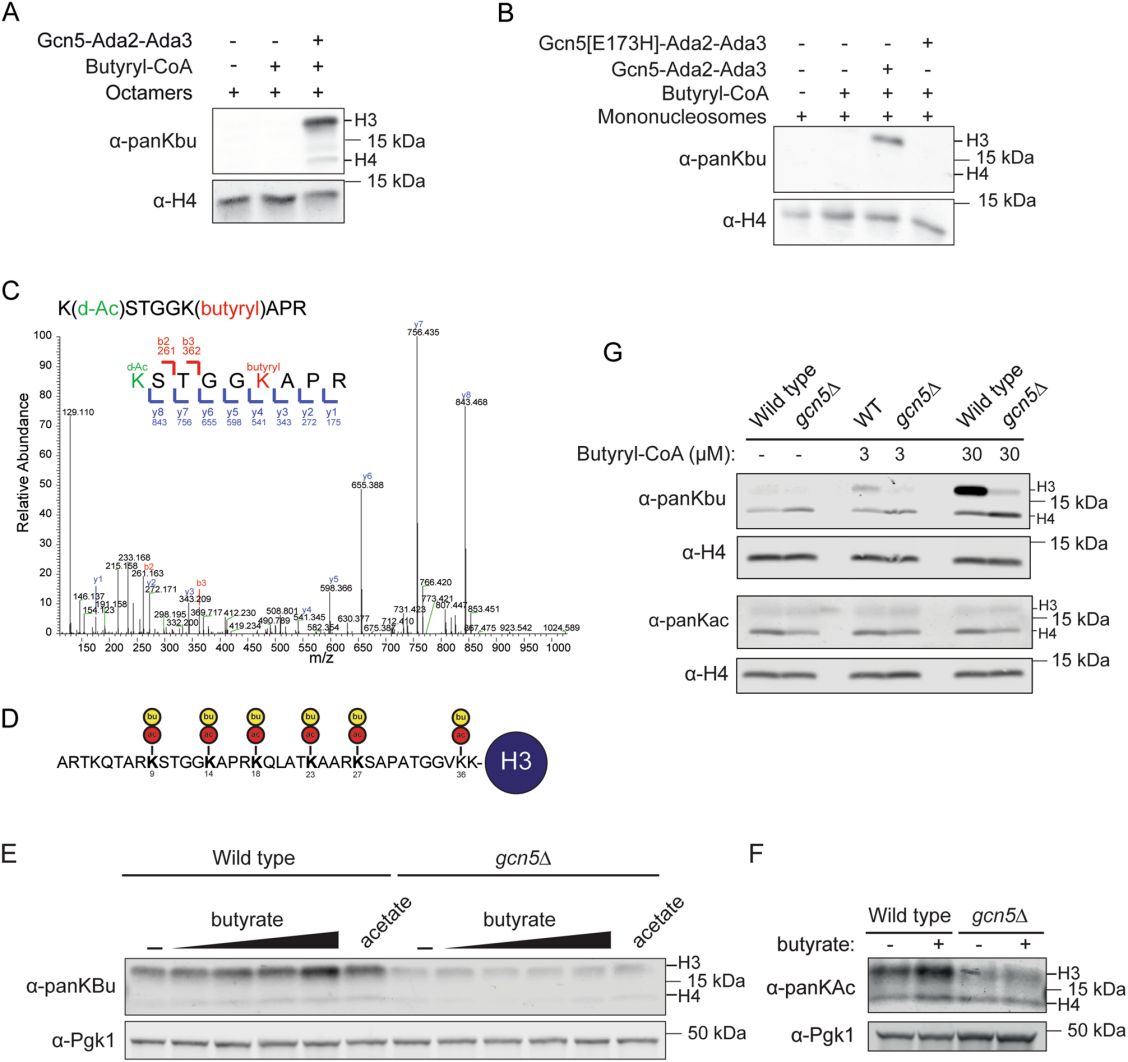
ADA complex displays histone butyryltransferase activity. (**A**,**B**) Western blot analysis of in vitro histone butyryltransferase assay with recombinant wild-type with (**A**) histone octamers and (**B**) mononucleosomes as a substrate. (**C**) Mass spectrometry spectrum of a peptide corresponding to a region spanning K9-R17 in histone H3 showing butyrylated K14 (red) and deuterated acetic acid (d) acetylations (green). (**D**) Schematic depicting residues 1–37 of H3 with acetylated (ac, red) and butyrylated (bu, yellow) lysine residues as identified by mass spectrometry. (**E**, **F**) Wild-type and *gcn5Δ* strains were treated with increasing concentrations of sodium butyrate (1, 2, 5, and 10 mM; pH 7.5) or sodium acetate (10 mM; pH 7.5) (**E**) or sodium butyrate (10 mM; pH 7.5) (**F**) for 3.5 h in the presence of 0.8 m sorbitol. Whole-cell extracts were immunoblotted with the indicated antibodies. (**G**) Western blot analysis using the indicated antibodies of nuclei isolated from wild-type and *gcn5Δ* strains. Nuclei were incubated with butyryl-CoA for 1 h.

Following our antibody validation and Epi-ID screen validation, we reasoned that the regulators identified in our Epi-ID screens may be regulators of histone acylation, particularly histone acetylation. To discriminate between these possibilities, we further investigated the ADA complex, one of the strongest common hits in the Epi-ID screens. We previously described a role for this complex in histone crotonylation in vitro and in vivo^13^. Here, we examined if Gcn5 is a writer for succinylation and butyrylation as well, as was previously described for hGCN5 (KAT2A)^20^. To avoid antibody specificity issues, we tested whether Gcn5 can directly succinylate and butyrylate histone residues in vitro. In these histone acyltransferase assays, we used previously purified ADA complex^13^, acyl-CoA and either histone octamers or mononucleosomes as a substrate. The core ADA complex (Gcn5, Ada2 and Ada3) was used since Gcn5 alone shows weak acetylation and crotonylation activity towards nucleosomes^13, 38, 42^. Since we used one type of acyl-CoA (succinyl-CoA or butyryl-CoA) as a donor, this generated unique acyl marks on histones and avoids misinterpretation of acylations in subsequent detection by western blot analysis. However, in the succinyltransferase assays, we observed strong non-enzymatic activity of succinyl-CoA with the octamers and nucleosomes (Supplementary Fig. 3), likely due to succinyl-CoA being highly reactive at physiological pH^43^. Although, surprisingly, auto-reactivity was not observed in a study in which KAT2A (hGcn5) was identified as a novel succinyltransferase^20^, this auto-reactivity prevented us from further examining ADA’s succinyltransferase activity. In contrast, we observed robust butyryltransferase activity for ADA towards both histone octamers and nucleosomes (Fig. 7A,B). This activity was fully dependent on Gcn5’s catalytic site, the glutamic acid on position 173 (Fig. 7B). Next, we investigated which lysines were butyrylated by ADA. To this end, we subjected our in vitro modified mononucleosomes to mass spectrometry analysis. We observed butyrylated residues at positions 9, 14, 18, 23, 27 and 36 on histone H3 (Fig. 7C,D and Supplementary Fig. 4). This is in concordance with previous findings showing that ADA targets the same lysines for acetylation and crotonylation^13^. Since the ADA complex can regulate histone crotonylation following the addition of crotonate to yeast cells^13, 14^, we investigated if ADA could also regulate histone butyrylation levels following the addition of extracellular butyrate. While butyrate indeed increased histone butyrylation, also acetylation levels were increased (Fig. 7E,F). Thus, butyrate, in contrast to crotonate, may contribute to acetylation by serving as a donor during acetyl-CoA formation and/or by functioning as an HDAC inhibitor^44, 45^. To circumvent this issue, we tested whether butyryl-CoA could induce butyrylation in a manner dependent on Gcn5 activity. Since acyl-CoAs are membrane impermeable, but transport is possible through nuclear pores^46^, we isolated nuclei from wild-type and *gcn5* mutant cells and incubated these nuclei with butyryl-CoA. Treatment with butyryl-CoA led to a strong increase in the histone butyrylation levels in wild-type, but not ﻿*gcn5* mutant cells, indicating Gcn5 is required for histone butyrylation (Fig. 7G). Thus, we identified the ADA complex as a novel writer of histone butyrylation in vitro and in vivo. These findings reveal broad activities of the ADA complex in generating various histone acylations by targeting the same lysines in H3.

## Conclusion and discussion

Here, we present a new application of the Epi-ID technology to study acylation modifications on chromatin. We performed four genome-wide Epi-ID screens to identify genes that affect acetylation, crotonylation, succinylation and butyrylation. Remarkably, the screens showed a high degree of correlation and the identified genes affecting histone acylation were virtually identical. Further studies revealed antibody cross-reactivity in various assays. In fact, the limited recognition of acylation by these antibodies, combined with the vast abundance of acetylation on chromatin, makes that the pan-K-acyl-recognizing antibodies mostly recognize histone acetylation in vivo. This suggests that the Epi-ID screens mainly served as a read-out for histone acetylation. Follow-up experiments on hits from the Epi-ID screen validated potential new regulators of histone acetylation. In addition, we uncovered that Gcn5, the catalytic subunit of the ADA complex, possesses butyryltransferase activity. This is in line with previous work suggesting that human Gcn5 has a similar activity, and expands ADA’s capacities as a writer complex^11, 20, 47^.

Antibody cross-reactivity has been widely observed in epigenetic research, especially in distinguishing different histone methylation states^48–50^. Moreover, antibody cross-reactivity is suggested to be a major factor in the “reproducibility crisis” plaguing science^51^. Here, we identified the cross-reactivity of various pan-K-acyl antibodies, which was heavily affected by differences in abundance between histone acetylation and the other acylation modifications in vivo. Initial dot-blot validation experiments suggested various degrees of specificity of these antibodies against crotonylation, succinylation or butyrylation marks, ranging from no off-target binding to moderate off-target binding^5, 31, 35, 52^. Indeed, expression of a deacetylation-deficient HDAC1 mutant which selectively removed crotonylation marks, only affected crotonylation but not acetylation levels after western blot or immunofluorescence analysis, indicating no acetylation recognition of the batch of pan-K-crotonylation antibody used in this study^15^. However, we report that multiple batches of pan-K-crotonyl and pan-K-butyryl antibodies were unable to distinguish between lysine crotonylation and butyrylation, suggesting cross-reactivity of these antibodies (Figs. 2 and S2). Indeed, other recent reports also indicated cross-recognition by several of these antibodies^21, 53^. These results by us and others show that the cross-reactivity of these antibodies with different acylation substrates could suffer from batch-to-batch differences. Therefore, validation with both dot blot and appropriate competition assays of each batch of antibody is recommended prior to initiating experiments.

Acetyl-BSA competed with all non-acetyl acylation antibodies in various assays, suggesting these antibodies cross-react with acetylation in vivo*.* Why a relatively weak binding to acetylation is responsible for the bulk of the signal, e.g. in ChIP experiments, can be explained by the relative abundance of the different acylation modifications. The abundance of lysine acetylation in HeLa cells far exceeds the amount of either histone crotonylation or butyrylation (19% vs 0.07% respectively)^21^. In fact, the abundance of histone acetylation, crotonylation and butyrylation appears to be in direct relation with the abundance of their metabolic precursors acetyl-CoA, crotonyl-CoA and butyryl-CoA^21^. We propose that the ~ 300-fold excess of acetylation is siphoning away most of the non-acetyl acylation antibody binding. Under such conditions, even a highly specific antibody may not sufficiently recognize a sparsely present antigen. Consequently, due to the vast overabundance of acetylation, the seemingly minor cross-reactivity of the crotonylation, succinylation or butyrylation antibodies results in substantial aspecific signals. Histone peptide microarrays have often successfully been used to assess antibody specificity, yet they do not consider the relative abundance of the epitope in vivo. The incorporation of acetylation control competitors is, therefore, an indispensable step in the validation of these antibodies in vivo. Our results suggest that only through insight in abundance provided by e.g. mass spectrometry-based proteomics of histone PTMs, and by including off-target competitors in the validation process, it is possible to predict if off-target recognition of antibodies poses a threat to the interpretation of data. Besides the pan-K-acyl antibodies, there are also several histone acyl-K-specific antibodies in use that we did not include in our validation. Based on our validation experiments and findings by others^21, 53^, we would, however, urge caution in the use of all non-acetyl acylation antibodies and the interpretation of data generated by these antibodies when support by additional antibody-independent experiments is lacking.

Our results strongly indicate cross-reactivity in batches of non-acetyl antibodies. Consequently, our Epi-ID screens mostly identified regulators of histone acetylation. To validate findings from the Epi-ID screens, we performed ChIP-qPCR for various factors and confirmed that loss of *GCN5, MET7*, *CLB3* and *MTF1* reduces, while loss of *HDA1, HDA2, RPD3* and *MTF1* increases the acylation levels locally at the barcoded *HO* locus. Moreover, western blot analysis revealed that loss of *GCN5* and *MET7,* but not that of *CLB3,* also reduces chromatin acetylation globally. Loss of *CLB3* may only affect specific regions, including the barcoded *TEF* promoter at the *HO* locus, leaving global acylation levels largely unaffected in the *clb3*Δ strain. Local loss of acetylation of promoter regions could explain the decrease in KanMX expression in *CLB3* mutants. Finally, loss of *RPD3,* and *RAD26,* led to an increase in global acetylation, albeit that the effect was moderate for *RAD26*. Thus, Epi-ID has the potential to identify known and unanticipated factors (*MET7, CLB3, MTF1,* and *RAD26*) that regulate histone acetylation, and possibly other histone acylations. However, unlike HATs and HDACs, these factors may not act directly on histone acylation, but potentially through their function in mitochondrial regulation (*MTF1* and *MET7*)^54, 55^, cell cycle progression (*CLB3*)^56, 57^ or transcription/DNA repair (*RAD26*)^58^. Since acetyl-CoA abundance is highly dependent on mitochondrial function^59, 60^, loss of Mtf1 (Mitochondrial RNA polymerase specificity Factor 1) or Met7 (Methionine requiring 7), the latter of which is involved in maintenance of mitochondrial DNA^61^, can potentially lead to decreased histone acetylation levels due to loss of acetyl-CoA production in dysfunctional mitochondria. Clb3 (Cyclin B3) is not known to affect histone acetylation directly. We therefore speculate that Clb3 may impact histone acetylation (and consequently transcription) indirectly by affecting cell cycle progression, a process known to impact cellular acetylation levels^62^. Finally, loss of Rad26 impairs transcription-coupled nucleotide excision repair of endogenous DNA lesions. These lesions may in turn become repaired by the alternative global genome nucleotide excision repair pathway, which has been associated with hyperacetylation of chromatin, possibly explaining the increased acetylation levels in cells lacking *RAD26*^63^*.* Further investigation of these and other factors affecting only a single barcode region, could lead to the identification of regulators that impact more specialized genomic regions, most notably promoters and terminators. Consequently, the Epi-ID dataset can also serve as a starting point for follow-up studies that aim to unravel the mechanistic role of such factors in regulating histone acetylation and beyond.

Considering the lack of specificity of the pan-K-acyl antibodies, we hypothesized that the regulators identified in our Epi-ID screens are either regulators of histone acetylation or histone acylation. To differentiate between these possibilities, we investigated the ADA complex, one of the strongest common hits in the Epi-ID screens, for succinylation and butyrylation activity. We showed that the ADA complex is a writer for butyrylation, in addition to it being a writer of acetylation and crotonylation. This finding is consistent with the idea that a subset of acetylation writers and erasers possess broad (de)acylation activities^12–15, 17, 20, 64^. The regulation of these activities is linked to the metabolic state of the cell^10, 19–22^. For example, crotonylation by Gcn5, Esa1 or p300 can be modulated by the addition of crotonate to the cells, leading to various transcriptional changes^13, 14^. However, how and if butyrate affects butyrylation and transcription through Gcn5, or other writers, is still unclear. Since we observed that butyrate can also contribute to acetylation, this complicates studying ADA’s transcriptional effect through butyrylation. Therefore, further studies are required to better understand the function of Gcn5 in butyrylation, transcription and its link to metabolism.

## Methods

### Yeast strains, cell lines, plasmids and antibodies

HeLa cells were STR genotyped and a kind gift of the department of Chemical and Cell Biology of the Leiden University Medical Center. HeLa cells were cultured in 5% CO_2_ at 37 °C in DMEM (Dulbecco’s modified Eagle’s medium) supplemented with 10% fetal calf serum (Bodinco B.V.) and antibiotics. The wild-type yeast strain BY4741, in which the KanMX marker was inserted at the *HO* gene, was used for all experiments (genotype: *MAT*a *can1Δ-STE2pr-Sphis5 lyp1Δ his3Δ1 leu2Δ0 ura3Δ0 met15Δ0 ho::(Barcoder001)KanMX* (Fig. 5A)^23^*.* Yeast libraries were crossed using a RoToR from Singer Instruments and synthetic genetic array (SGA) technology^27^. The collection of barcoded yeast mutants was generated by crossing 1140 Barcoder strains^29^ to the *MAT*α NatMX knock-out^27^ and *MAT*α NatMX DaMP collections^28^ of yeast mutants. Validation experiments were performed with mutants from the library collections, which were verified by PCR. The *RPD3﻿* containing plasmid was constructed by amplifying the *RPD3* locus from genomic DNA and cloning into the pRS316 ARS/CEN vector. Mutant *RPD3* was generated by replacing AGG on positions 146–148 by VRPP by site-directed mutagenesis. RPD3-VRPP F (5ʹ-GTGATGTTGCTGTCAACTATGTTCGTCCGCCCTTGC ATCATGCAAAAAAATCGG-3ʹ), RPD3-VRPP R (5ʹ-CCGATTTTTTTGCATGATGCAAGGGC GGACGAACATAGTTGACAGCAACATCAC-3ʹ), Antibodies used were anti-pan-K-acetylation (PTM-105; PTM-Biolabs), anti-pan-K-crotonylation (PTM-501; PTM Biolabs), anti-pan-K-butyrylation (PTM-301; PTM Biolabs), anti-pan-K-succinylation (PTM-419; PTM Biolabs), Acetyl-Histone H3 (Lys9) (PTM-112; PTM Biolabs), anti-tubulin (cloneDM1A, T6199; Sigma), anti-Pgk1 (#459250; Invitrogen), anti-H4 (ab10158; Abcam) and anti-H3 (ab1791; Abcam).

### ChIP-qPCR

ChIP was performed as previously described^65^, except that for competition assays antibodies and either unmodified or acylated BSA were simultaneously added to each extract and incubated for two hours. In high BSA conditions 15 μg of BSA was added (6 μg BSA per μl antibody) to each extract, while in low BSA conditions 1.25 μg of BSA was added (0.5 μg BSA per μl of antibody) to each extract. Input and immunoprecipitated DNA was purified and analyzed by quantitative (q)PCR using primers:

- HOpro ChIP-qPCR F (5ʹ-GAAGCTTGTTGAAGCATGATGAA-3ʹ),
- HOpro ChIP-qPCR R (5ʹ-TTGCTGCTTATGAGGATATGGATTT-3ʹ),
- HOterm ChIP-qPCR F (5ʹ-GAGTAGAAATACGCCATCTCAAGATACA-3ʹ),
- HOterm ChIP-qPCR R (5ʹ-GGAAAGTTGATCAAGACCCAATAATAA-3ʹ),
- Telomere 6R ChIP-qPCR F (5ʹ- GGCTGGACTACTTTCTGGAATAGC-3ʹ),
- Telomere 6R ChIP-qPCR R (5ʹ- GAACTGTGCATCCACTCGTTAGG-3ʹ).

The HOpro amplicon is 150 bp and located 43 bp upstream of the UpTag barcode. The HOterm amplicon is 97 bp and located 120 bp downstream of the DownTag barcode.

### Epi-ID

Epi-ID was performed as described^23, 25^, except that for ChIP the following antibodies were used: anti-pan-K-acetylation (PTM-105; PTM-Biolabs), anti-pan-K-crotonylation (PTM-501;PTM Biolabs), anti-pan-K-butyrylation (PTM-301; PTM Biolabs), anti-pan-K-succinylation (PTM-419; PTM Biolabs) and anti-H3 (ab1791; Abcam). Deep-sequencing was performed on a single-end flowcell Illumina Hi-Seq2500.

### In vitro modification of BSA

BSA (BSAV-RO, Roche) was dissolved at 1 mg/ml in saturated NaHCO_3_. Acetic anhydride (320102, Sigma-Aldrich), crotonic anhydride (130974, Sigma-Aldrich), butyric anhydride (19270, Sigma-Aldrich) and succinic anhydride (239690, Sigma-Aldrich) were dissolved at 1 M in acetonitrile and added to the BSA solution at a final concentration of 25 mM at 0 °C. The mixtures were slowly warmed to room temperature and placed on a shaking incubator overnight.

### Mass spectrometry (MS) analysis of modified BSA

Gel slices containing BSA and modified (acetyl-, crotonyl-, butyryl-, or succinyl-) BSA protein were subjected to reduction with dithiothreitol, alkylation with iodoacetamide, acetylation with deuterated acetic acid anhydride and digestion with trypsin using Proteineer DP digestion robot (Bruker). Lysine residues were acetylated to block cleavage by trypsin. Deuterated acetic acid anhydride was used to discriminate between genuine acetylation and acetylation by the blocking agent. MS analysis of the tryptic BSA peptides was performed as published previously^13^. Methionine oxidation, d6-acetylation, acetylation, butyrylation, succinylation and crotonylation of lysines were set as variable modification. Carbamidomethyl on cysteine was set as a fixed modification. The false discovery rate at the peptide level was set to 1% and the Mascot ion threshold score to 35. The number of PSMs found for lysine-containing peptides of BSA are given in Table 1. Mass spectrometry data have been uploaded to the PRIDE repository (accession number #478371).

### Dot-blot assay

Serial dilutions of BSA and modified (acetyl-, crotonyl-, butyryl-, or succinyl-) BSA protein were spotted on nitrocellulose blots. After drying for 1 h, blots were incubated with blocking buffer (MB-070, Rockland) in PBS. Primary antibody was incubated in blocking buffer for 1 h at 4 °C. Washing was performed with 0.1% Tween20 in PBS. LI-COR Odyssey secondary antibody (1:10,000) was incubated for 30 min in blocking buffer at room temperature. LI-COR Odyssey Imager (Biosciences) was used for membrane scanning. Original uncropped blots are shown in Fig. 2.

### Western blot analysis

Yeast whole-cell extraction and western blot analysis were performed as described^13^. HeLa whole-cell extracts were prepared by dissolving and boiling cells in 2X Laemmli. After SDS-PAGE and transfer to membranes, proteins were stained with Ponceau. For competition assays, membranes were stained with Ponceau S and cut in between individual lanes. Next, antibody and either unmodified or acylated BSA, or antibody and either unmodified or acetylated (K9, 14) H3 peptide derived from human histone H3 (1–21) (#H12-58 and H12-358-500; SignalChem) were incubated simultaneously for 1 h after which membranes were developed as described^13^. Original uncropped blots are shown Supplementary Figure 5.

### Isolation of yeast nuclei

Yeast nuclei were isolated from 50 ml mid-log growing cells. Cell pellets were washed once in water, resuspended in 50 ml 100 mM Hepes–KOH pH 9.4, 10 mM DTT and 0.8 M sorbitol (pH 9.5) and incubated with shaking at 30 °C Celsius for 15 min. Cell pellets were resuspended in spheroplast medium containing 1× YNB, 2% glucose, 1× amino acids, 1 M Sorbitol and 20 mM Hepes (pH 7.2) supplemented with zymolyse (final concentration 2.5 μl/ODU) for 60 min at 30 °C to degrade the cell wall. Nuclei were isolated by resuspending yeast spheroplasts in cold nuclear isolation buffer (NIB) (15 mM Tris–HCL [pH 7.5], 60 mM KCl, 15 mM NaCl, 5 mM MgCl_2_, 1 mM CaCl_2_, 250 mM sucrose, 0.1% NP-40) supplemented with protease inhibitors leupeptin and pepstatin for 5 min. Nuclei were pelleted followed by two washes using NIB buffer without NP-40 for 20 min each. Nuclei were resuspended with the indicated concentrations of acetyl-CoA and butyryl-CoA in 200 μl NIB buffer without NP-40 and incubated for 1 h at 37 °C. After incubation, reactions were inhibited by adding sample buffer and analyzed by western blot.

### Nucleosome reconstitution

Purified histones and mononucleosomes were isolated as described^66, 67^.

### In vitro acylation assays

Histone acylation assays were performed with previously purified recombinant wild-type Gcn5-Ada2-Ada3 complex and catalytic-dead Gcn5(E173H)-Ada2-Ada3 complex^13^ using 0.5 µM mononucleosomes and 30 µM acetyl-CoA, 300 µM succinyl-CoA or 300 µM butyryl-CoA in reaction buffer (50 mM Tris–Cl pH 7.5; 100 mM NaCl; 1 mM EDTA, 1 mM DTT) in a final volume of 20 µl. After 1 h at 30 °C, reactions were inhibited by adding sample buffer and analyzed by western blot. For MS analysis, samples were separated on SDS-PAGE and the gel was stained with Coomassie Brilliant Blue. Bands corresponding to H3 were excised from the gel for MS analysis, as described previously^13^.

### Immunofluorescence analysis

HeLa cells were grown on 18 mm coverslips. Cells were fixed with 4% formaldehyde in PBS for 15 min at room temperature (RT), post-extracted with 0.5% Triton-X100 (T8787, Sigma-Aldrich) in PBS. Cells were treated with PBS + (PBS with 5 g BSA/L, 1.5 g glycine/L) 10 min to block unreacted aldehyde groups. Antibody steps and washes were in PBS+ . Primary antibodies were incubated for 1 h at room temperature and incubated with BSA or modified (acetyl-, crotonyl-, butyryl-, or succinyl-) BSA protein as competitor. Detection was done using goat anti-mouse or goat anti-rabbit Ig coupled to Alexa 488 or 555 (1:1500; Invitrogen Molecular probes). Samples were incubated with 0.1 μg/ml DAPI and mounted in Polymount. Images of the cells were taken with a Zeiss AxioImager D2 widefield fluorescence microscope equipped with a 40×, 63× and 100× PLAN APO (1.4 NA) oil-immersion objective and an HXP 120 metal-halide lamp used for excitation, and with the software ZEN 2012 (https://www.zeiss.com/microscopy/us/products/microscope-software/zen.html). Images were analyzed in ImageJ2 (https://imagej.net/ImageJ2) using an in-house developed, custom-built macro (available upon request) that enabled automatic and objective analysis of nuclear signal intensities. Briefly, cell nuclei were detected using minimal cross-entropy thresholding based on nuclear DAPI signal^68^. Intensities in channel 488 and 555 were measured in the complete nucleus, and background-subtracted based on the intensity outside the nuclear selection. Data were plotted using GraphPad Prism 7 (https://www.graphpad.com/scientific-software/prism/).

### RT-qPCR

Total RNA was isolated and genomic DNA was removed with the TURBO DNA-free Kit (Invitrogen). cDNA was prepared from total RNA using the GoScript Reverse Transcription System according to the manufacturer's protocol (Promega). qPCR was performed using GoTaq qPCR Master Mix (Promega) and analyzed by quantitative (q)PCR using primers: KANMX 5ʹ RT-qPCR F (5ʹ-CTTCCGACCATCAAGCATTTTAT-3ʹ), KANMX 5ʹ RT-qPCR R (5ʹ-GGATCGCAGTGGTGAGTAACC-3ʹ), ACT1 RT-qPCR F (5ʹ-ACGTTCCAGCCTTCTACGTTTCCA-3ʹ), ACT1 RT-qPCR R (5ʹ-TCGAAGTCCAAGGCGACGTAACAT-3ʹ). Expression levels were quantified using the 2−ΔΔCt method with *ACT1* as a reference gene. Specificity of primers was confirmed by melting curve analysis and tested for linear amplification.

### Statistical analysis

Correlation between Epi-ID datasets was calculated using Spearman correlation in RStudio (Version 1.3.1093). Statistical analysis of ChIP-qPCR and RT-qPCR was performed using two-way analysis of variance (ANOVA) with correction for multiple comparisons in Graphpad Prism 9 software (https://www.graphpad.com/scientific-software/prism/) to test the differences between a normalized wild-type and mutant strain. Significance is indicated as: ***p < 0.001, **p < 0.01, *p < 0.05. ns, not significant. All error bars represent the s.e.m..

## Supplementary Information

Supplementary Information 1.

Supplementary Information 2.

## Data Availability

All data generated for Epi-ID during this study are included in this published article and Supplementary Information 2.
