# Supplementary material for: Epigenetics Identifier screens reveal regulators of chromatin acylation and limited specificity of acylation antibodies
Source: Sci Rep. 2021 Jun 17;11:12795. doi: 10.1038/s41598-021-91359-0 (PMC8211816; doi:10.1038/s41598-021-91359-0)
Supplement: Supplementary file 1 — Supplementary Information 1. [file 41598_2021_91359_MOESM1_ESM.pdf]

Supplementary Information 1

Epigenetics Identifier screens reveal regulators of chromatin acylation and limited specificity of acylation antibodies

Leonie Kollenstart, Sophie C. van der Horst, Kees Vreeken, George M.C. Janssen, Fabrizio Martino, Hanneke Vlaming, Peter A. van Veelen, Fred van Leeuwen and Haico van Attikum

This file contains Supplementary Figures 1-5

Kollenstart *et al.*, Supplementary Figure 1

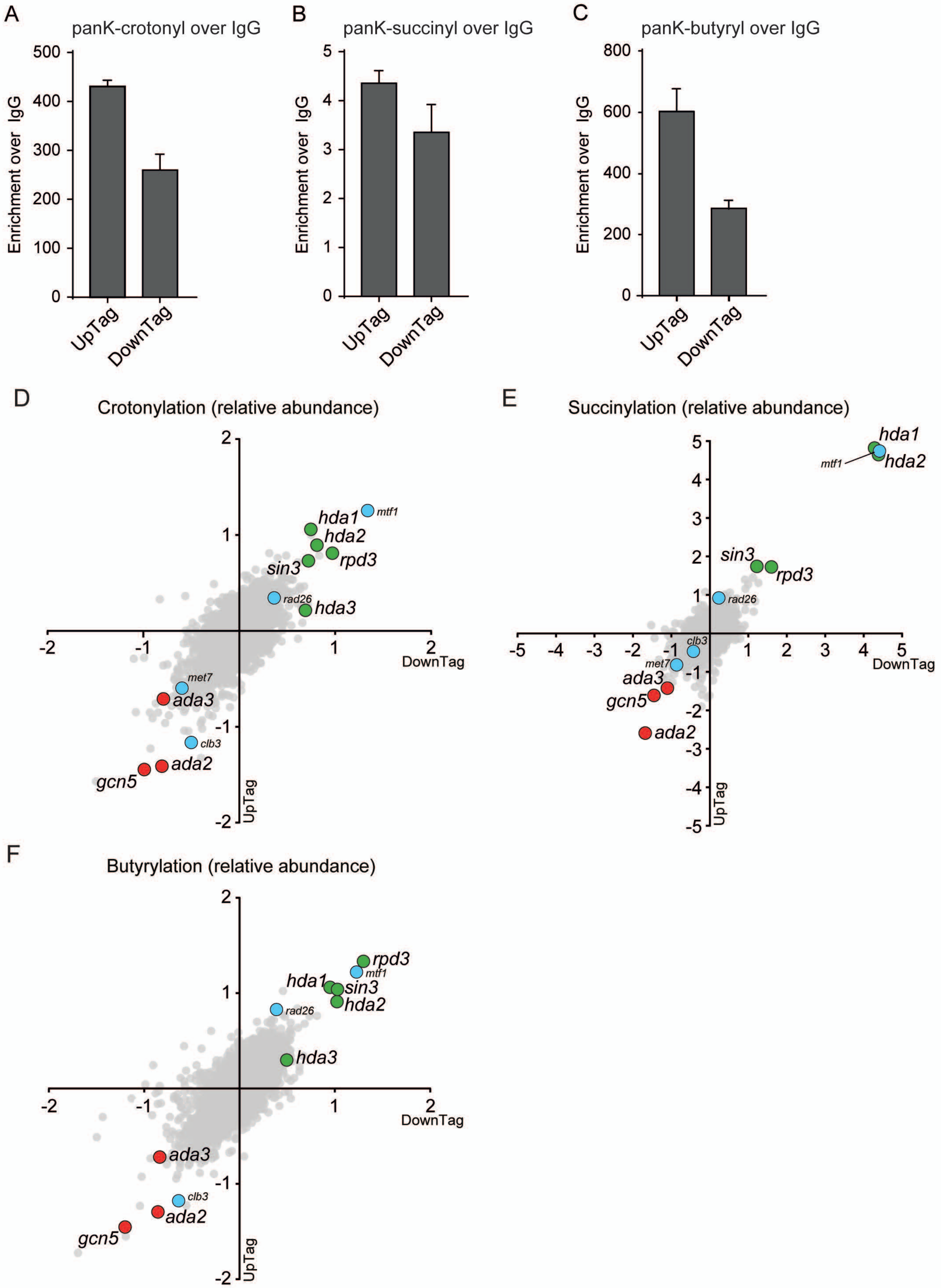

Supplementary Fig 1. Presence of histone acylations at UpTag and DownTag barcodes and Epi-ID results for crotonylation, succinylation and butyrylation

ChIP-qPCR analysis of (A) panK-crotonylation, (B) panK-succinylation and (C) panK-butyrylation at the UpTag and DownTag. Data represent the mean fold enrichment over an IgG control ChIP from three independent experiments + s.e.m. Values were normalized to WT for each primer set. Outcome of Epi-ID for (D) crotonylation, (E) succinylation and (F) butyrylation on approximately 6500 strains. Scatter plots show ChIP/Input ratios normalized to H3 abundance for UpTag versus DownTag. Averages of two independent Epi-ID screens are shown. Each dot represents a single mutant strain. Mutants of the ADA (red), RPD3 (green), HDA1 (green) complexes and newly identified mutants selected for follow-up (blue) are highlighted.

Kollenstart *et al.*, Supplementary Figure 2

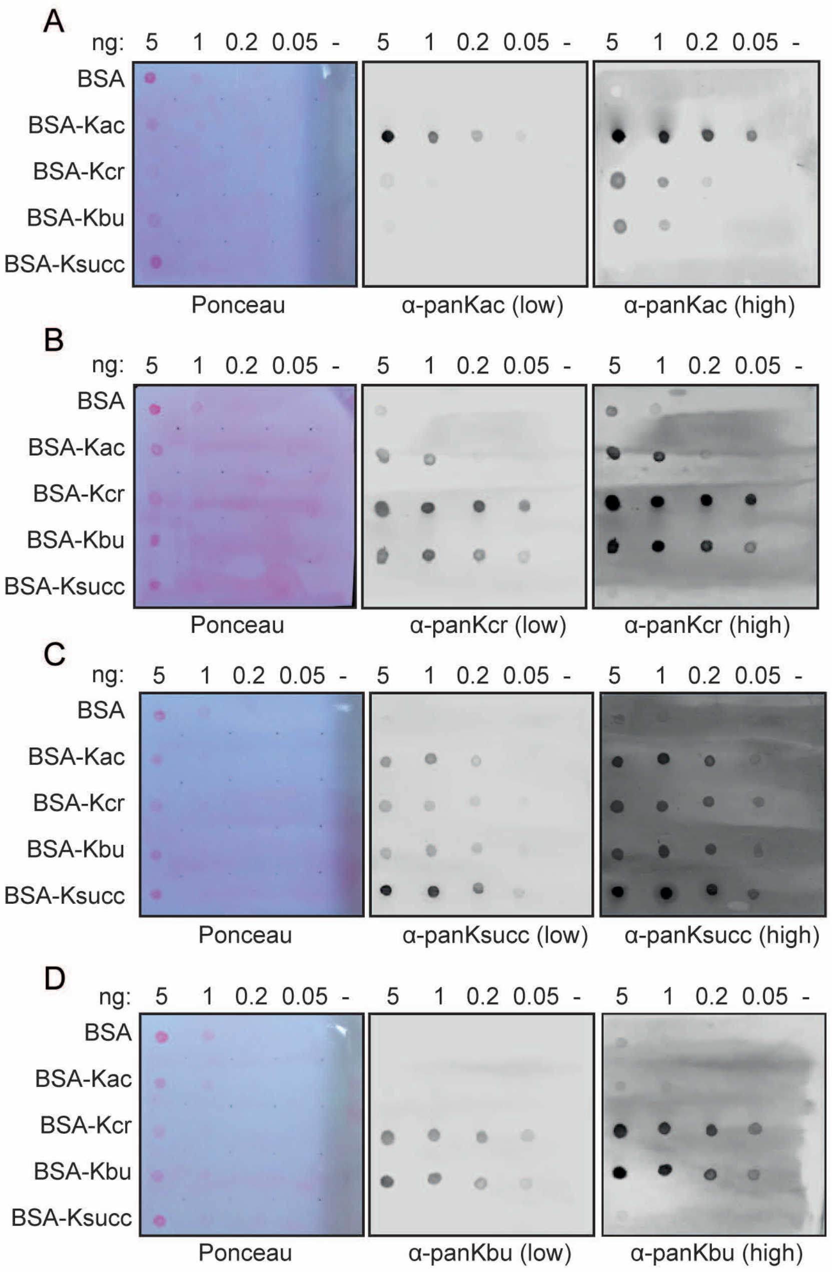

Supplementary Fig 2. Dot-blots of modified BSA to test pan-K-acyl antibody specificity.

(A-D) Immunoblot analysis of serially diluted unmodified and modified BSA using the indicated antibodies.

Kollenstart *et al.*, Supplementary Figure 3

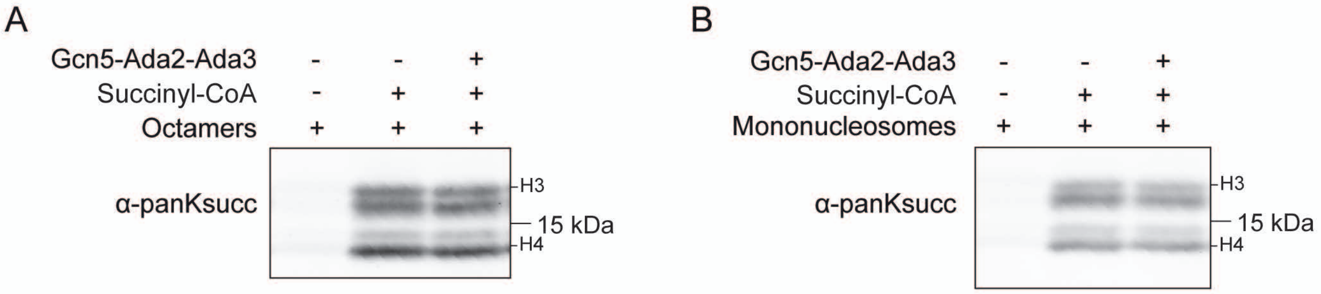

Supplementary Fig 3. Non-enzymatic succinylation of histones *in vitro*

Western blot analysis of *in vitro* succinyltransferase reactions performed with recombinant wild-type ADA complex on (A) histone octamers and on (B) mononucleosomes as substrates.

Kollenstart *et al.*, Supplementary Figure 4

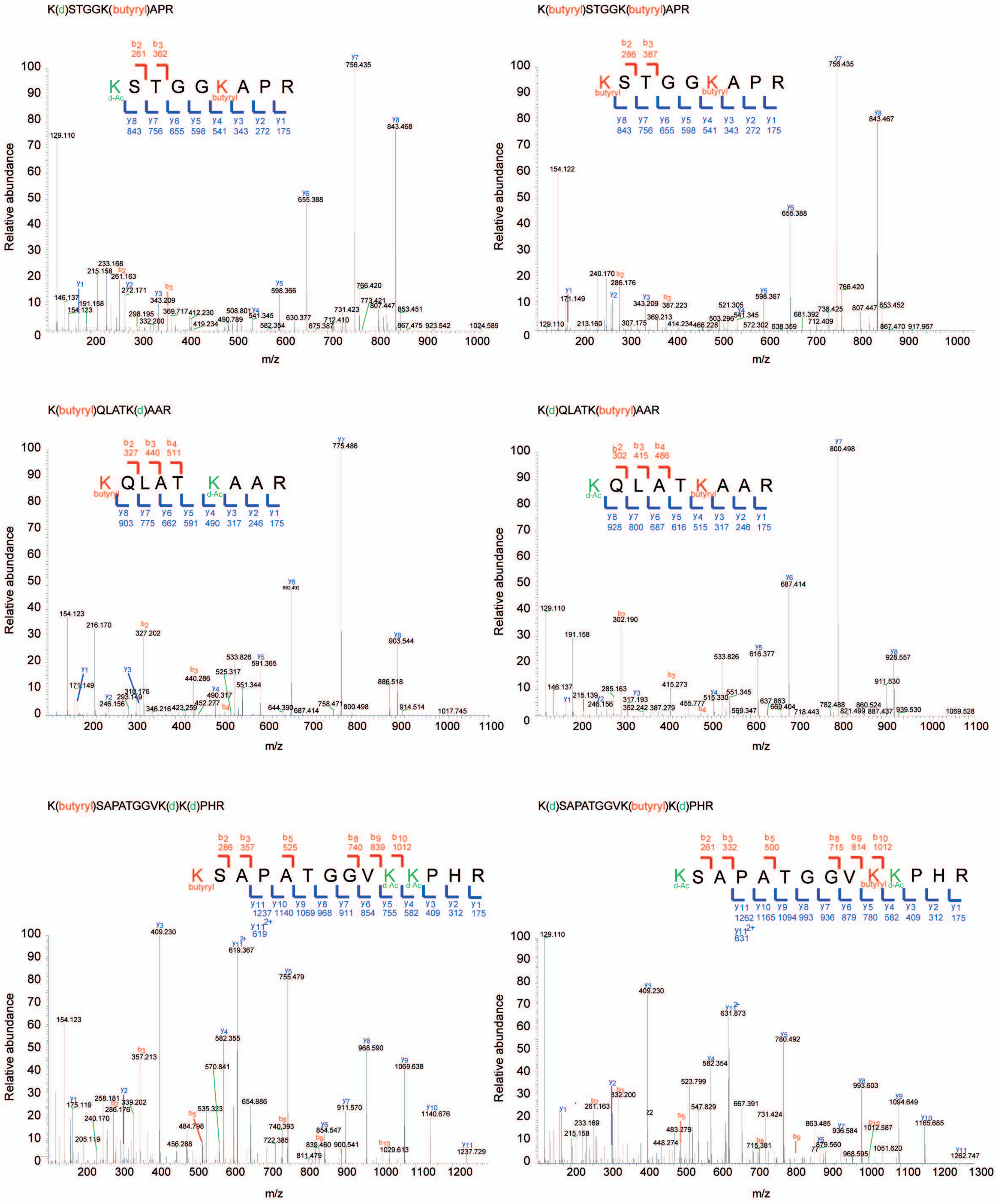

Supplementary Fig 4. MS/MS spectra of butyrylated peptides.

MS/MS spectra leading to the identification of all butyrylated peptides found on histone H3. Butyrylation by Gcn5-Ada2-Ada3 is shown in red while deuterated acetic acid (d) acetylations are shown in green.

Kollenstart *et al.*, Supplementary Figure 5

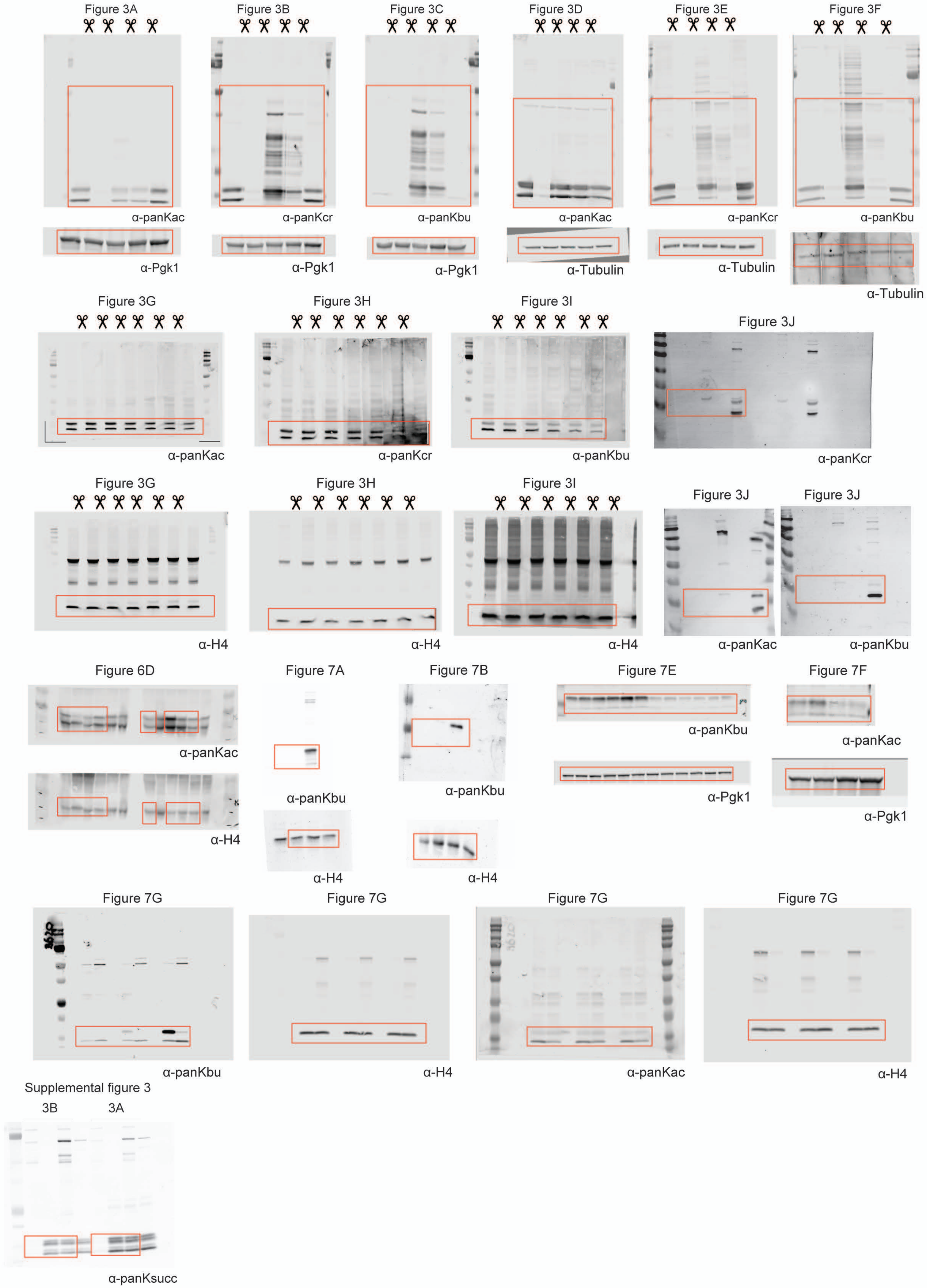

Supplementary Fig 5. Uncropped blots

Uncropped blots of all western blots presented in this manuscript. The scissors above the images from Figure 3A-3G indicate the lines where the blot was cut prior to incubation with the antibody. Red boxes demarcate the regions that are shown as cropped blots in the main or and supplementary figures. Since these images were all developed with the LI-COR Odyssey Imager, they have not all been scanned in their full length.
